# *Phellodendron *and *Citrus *extracts benefit joint health in osteoarthritis patients: a pilot, double-blind, placebo-controlled study

**DOI:** 10.1186/1475-2891-8-38

**Published:** 2009-08-14

**Authors:** Julius Oben, Ebangha Enonchong, Shil Kothari, Walter Chambliss, Robert Garrison, Deanne Dolnick

**Affiliations:** 1 Laboratory of Nutrition & Nutritional Biochemistry, Department of Biochemistry, University of Yaounde I, Cameroon; 2 Gateway Health Alliances, Inc., 4769 Mangels Blvd, Fairfield, CA 94534 USA; 3 University of Mississippi, University, MS 38677 USA; 4 Next Pharmaceuticals, 360 Espinosa Road, Salinas, CA 93907 USA

## Abstract

**Background:**

The objective of this clinical study was to assess the potential benefit of a dietary supplement, NP 06-1, on joint health in overweight and normal weight adults diagnosed with osteoarthritis.

**Methods:**

An 8-week placebo-controlled, randomized, double-blind study was conducted with four groups comparing the effects of NP 06-1 to placebo on overweight and normal weight subjects diagnosed with primary osteoarthritis of the knee. NP 06-1 (a combination of two botanical extracts; *Phellodendron amurense *bark and *Citrus sinensis *peel) or matching placebo were given in a dose of two capsules (370 mg each) twice daily. The outcome measures were the Lequesne Algofunctional Index (LAI) for joint pain and movement as well as biomarkers of inflammation (C-reactive protein [CRP] and erythrocyte sedimentation rate [ESR]).

**Results:**

Eighty (80) subjects were enrolled and 45 subjects completed the study. No serious adverse events were reported. The mean total LAI scores at baseline for the four groups ranged from 11.4 to 12.4 (SD 1.2 to 2.4). Treatment for 8 weeks resulted in a statistical improvement in the LAI score in the overweight treatment group compared to placebo (6.3 ± 2.3 vs 11.8 ± 1.5; p < 0.0001). At 8 weeks, a similar result was observed in the normal weight groups (7.7 ± 1.4 vs 9.9 ± 0.9; p < 0.0001). There was a reduction in CRP levels with treatment in the overweight treatment group at 8 weeks (-0.62 ± 0.2; 49%) compared to baseline (p < 0.001) and to placebo (p < 0.001). For the normal weight participants, there were significant reductions in CRP compared to baseline, but not to the matched placebo group. Both overweight and normal weight treatment groups lost a significant amount of weight compared to their placebo groups. The overweight treatment group lost an average of 5% body weight after 8 weeks. There was no significant change in ESR in any of the groups.

**Conclusion:**

In this pilot study, NP 06-1 had beneficial effects on symptoms of osteoarthritis of the knee as measured using LAI scores and had anti-inflammatory effects as measured using CRP. Administration of NP 06-1 was also associated with weight loss, which may have been a contributing factor to the other benefits.

## Background

Osteoarthritis, the most common form of arthritis, is characterized by degradation of articular cartilage, which manifests as joint pain and reduced mobility. The origin of the disease is unknown but obesity, joint injury, metabolic diseases, bone and joint malfunctions, genetic factors and age have been implicated. Therapies for osteoarthritis include weight control, physiotherapy and pharmacological agents. Conventional drug treatments include analgesics, anti-inflammatory agents, disease modifying therapies, hyaluronic acid, intra-articular glucocorticoids and topical analgesic/anti-inflammatory agents [1]. More recently there has been a focus on nutritional support. Recent systematic reviews highlight the scientific evidence for potential nutritional and herbal preparations for those with osteoarthritis [2,3].

The subject of this study, NP 06-1 (Citrofen^®^, Next Pharmaceuticals, Salinas, CA), is a proprietary product consisting of a blend of extracts of *Phellodendron amurense *tree bark and *Citrus sinensis *(orange) peel standardized to berberine and polymethoxylated flavones, respectively.

Next Pharmaceuticals has previously conducted bioassay guided research on *Phellodendron amurense *tree bark resulting in a proprietary extract that has demonstrated analgesic and anti-inflammatory activity both *in vitro *and *in vivo*, in unpublished studies. Following this research, a product was developed (Nexrutine^®^) that provided relief from pain and/or inflammation associated with over-exertion (i.e. sore muscles, sore joints, stiff joints) and osteoarthritis of the knee in unpublished open-label clinical studies.

*Citrus sinensis *(orange) peel extracts contain bioflavonoids, including polymethoxylated flavones (PMFs). The latter compounds are known to be anti-inflammatory and to have antioxidant and hypolipidemic effects [4-6]. Next Pharmaceuticals has licensed US Patent Nos. 6,184,246 and 6,987,125 obtained by the USDA for actions described by PMFs.

NP 06-1 was formulated with the goal of combining the beneficial effects of both the phellodendron and orange peel extracts. The primary objective of this clinical study was to study the safety and efficacy of NP 06-1 in the management of joint pain and mobility caused by osteoarthritis of the knee. Secondary objectives were to study the effects on biomarkers related to inflammation. Both overweight and normal weight subjects were studied to determine whether or not there might be a difference in benefits to these two groups. A portion of this clinical study that examined the potential benefits from NP 06-1 on cardiovascular health has recently been published [7]. The published data showed that NP 06-1 had a favorable effect on lipid levels: lowering plasma triglycerides and LDL-cholesterol, as well as increasing HDL-cholesterol levels. NP 06-1 also had a favorable effect on blood pressure and fasting glucose levels (the latter only in the overweight group).

## Methods

### Study Design

The study was a placebo-controlled, randomized, double-blind study with four groups. Forty overweight and forty normal weight subjects were enrolled into either treatment or placebo groups, with twenty subjects in each group as shown in Table 1. Subjects were recruited via advertisements at the University of Yaounde I Teaching Hospital, the Djongolo Baptist Hospital and through the public media. The IRB of the Faculty of Medicine and Biomedical Sciences of the University of Yaounde in Cameroon approved the study and all subjects signed an informed consent. Dr. Julius Oben, Associate Professor of Nutritional Biochemistry at the University of Yaounde I, Cameroon was the principal investigator.

**Table 1:** Study Groups.

**Group #**	**BMI Category**	**Treatment**	**Enrollment (Completed)**
**OP**	Overweight*	Placebo	20 (13)

**OT**	Overweight*	Active	20 (14)

**NP**	Normal weight†	Placebo	20 (7)

**NT**	Normal weight †	Active	20 (11)

*BMI 25 kg/m^2 ^– 40 kg/m^2 ^†BMI 18.9 kg/m^2 ^– 24.9 kg/m^2^

The number of subjects initially enrolled is listed; with the number that completed the study in parenthesis.

To be included in the study, participants needed to be adult men and women, 25 to 60 years of age, diagnosed with primary osteoarthritis of the target knee using the American College of Rheumatology guidelines [8] by the treating physician and confirmed by the clinical investigator, and with a BMI between 25 kg/m^2 ^to 40 kg/m^2 ^(overweight groups) or 18.9 kg/m^2 ^to 24.9 kg/m^2 ^(normal weight groups).

The exclusion criteria were: morbid obesity BMI > 40 kg/m^2^, diagnosis of rheumatoid arthritis, joint replacement in either knee, unable to walk without assistance, enrollment in another clinical study in the past 6 months, pregnancy, active infection, autoimmune disease, AIDS, HIV, active hepatitis, active malignancy, and diabetes requiring daily insulin management.

Subjects were instructed to avoid taking analgesics for a 5 day period prior to enrollment and during the study. Subjects were also instructed to stay with their normal exercise and diet regimens.

### Study Products and Administration

NP 06-1 is a proprietary product of Next Pharmaceuticals, Salinas, California. It is a blend of *Phellodendron amurense *(Rupr.) [Rutaceae] tree bark extract standardized to a minimum of 50% berberine and *Citrus sinensis *(L.) Osbeck [Rutaceae] peel extract standardized to a minimum of 30% polymethoxylated flavones (PMF). Berberine and PMFs were chosen as representative chemical constituents because they have demonstrated biological activity. Next Pharmaceuticals has licensed US Patent Nos. 6,184,246 and 6,987,125 obtained by the USDA for biological activity of PMFs.

Subjects were allocated into groups using a random number table and instructed to take two NP 06-1 capsules (370 mg formula per capsule) or matching placebo (identical red two-piece hard shell capsules) with food in the morning and at night (4 capsules per day) for a total of 8 weeks.

### Study Variables

The study variables were: physical pain and joint movement as measured by the total score on the standardized LAI questionnaire [9], as well as biomarkers for inflammation: CRP and ESR. The LAI scale (algofunctional indices) is a validated instrument for evaluating therapies for osteoarthritis. The LAI score is the sum of the responses to 10 questions regarding pain/discomfort, maximum distance walked and activities of daily living. The subjects were asked to respond to each question using a 5 point Likert scale (0, 0.5, 1.0, 1.5 or 2.0; where 0 is without and 2 is with difficulty/discomfort). The LAI questionnaire was completed by each subject at baseline, at 4 weeks and at 8 weeks. Blood samples (8 ml) were collected by venous puncture at times 0, 4 and 8 weeks. Subjects were requested to come to the study center on the mornings of each site visit after a 12-hour fast. CRP was measured using the Dade Turbitimer. ESR was measured using the Westergren method. Body weight was determined in 12-hour fasted subjects using a Tanita™ scale and height was measured using a stadiometer.

### Safety Assessment

Subjects were given three emergency telephone numbers to contact during the conduct of the study, if they had any adverse events or other concerns related to the study. Each subject was interviewed during site visits to solicit information on possible adverse effects they might have encountered. Participants were instructed to inform the investigator, if the reason for dropping out of the study was due to adverse effects.

### Statistical Analysis

Data were analyzed using SAS version 9.2 (SAS Institute, 1999). Descriptive statistics were run for each group (Placebo and NP 06-1) and each time point (baseline, 4 weeks and 8 weeks). Initially, an analysis of variance model was used to determine whether or not the groups and times differed in baseline scores. No differences were found. An analysis of variance model, with repeated measures, was used to compare changes of physiological measures over the trial period and between groups. First, we examined models with all 2-way and 3-way interactions. Since the 3-way interactions were found significant, we examined each weight group separately. For each weight group, we used an analysis of variance model with treatment (Placebo vs and NP 06-1) as a fixed effect and time (4 weeks vs 8 weeks) as a repeated measure to determine the effects of NP 06-1.

## Results

Eighty (80) subjects were enrolled in the study and randomized into four groups designated OP (Overweight/Placebo), OT (Overweight/Treatment), NP (Normal weight/Placebo) and NT (Normal weight/Treatment) (Table 1). Forty five (45) subjects completed the study and the reasons given by the dropouts are given in Table 2. The dropouts were fairly evenly distributed among the groups, with the exception of Group NP which lost nearly twice as many participants as the other groups. The majority of Group NP dropouts cited no improvement in their condition as their reason for dropping out. There is no indication that their demographics were different from those who stayed in the study. No serious adverse events were reported by any of the dropouts or by those who completed the study. The following results were compiled using data from those who completed the study.

**Table 2:** Reasons given by Subjects for dropping out of the Study.

**Group #**	**Number of Subjects**	**Reason for drop out**
**OP**	2	Reported no improvement in their condition

	1	Nausea and vomiting; Malaria attack

	1	Moved out of town

	3	No reason given

		

**OT**	1	Reported improvement as too slow

	1	Tested positive for hepatitis

	1	Moved out of town

	3	No reason given

		

**NP**	7	Reported no improvement in their condition

	1	Nausea

	1	Started weight management program

	1	Started fasting and stopped treatment

	3	No reason given

		

**NT**	1	Malaria attack

	1	Nausea

	1	Started fasting and stopped treatment

	6	No reason given

### LAI Scores

The mean total LAI scores at baseline for the four groups ranged from 11.4 to 12.4 (standard deviations 1.2 to 2.4) with no statistical differences between them (p = 0.43) (Table 3). Comparison of the overweight groups (OT vs. OP) showed a significant decrease in LAI scores in the treatment group compared to the placebo at 4 weeks (8.2 ± 2.7 vs 11.7 ± 1.7) and at 8 weeks (6.3 ± 2.3 vs 11.8 ± 1.5) (both p < 0.0001) (Table 4). For Group OT, there were statistically significant decreases in LAI scores at 4 weeks (-3.5 ± 2.2) and 8 weeks (-5.4 ± 2.2) compared to baseline (both p < 0.0001) (Table 3). There was also a difference between the 4 and 8 week time periods (p = 0.002). In contrast there were no statistically significant changes from baseline for Group OP.

**Table 3:** LAI data.

**Group**	Baseline	4 weeks	8 weeks	Δ 0–4 wks	Δ 0–8 wks
**OP**	12.4 ± 1.3	11.7 ± 1.7	11.8 ± 1.5	-0.7 ± 1.6	-0.6 ± 1.7

**OT**	11.7 ± 1.5	8.2 ± 2.7	6.3 ± 2.3	-3.5 ± 2.2*	-5.4 ± 2.2*

					

**NP**	11.7 ± 2.4	10.3 ± 1.5	9.9 ± 0.9	-1.4 ± 2.8*	-1.9 ± 2.1*

**NT**	11.4 ± 1.2	7.6 ± 1.9	7.7 ± 1.4	-3.7 ± 2.1*	-3.7 ± 1.7*

Values at baseline, 4 weeks and 8 weeks are listed as means ± standard deviation. Mean changes (Δ) ± standard deviation after 4 weeks and 8 weeks compared to baseline are listed in the last two columns. A negative value indicates a decrease in value. Values* are significant comparisons (<0.05) based on analysis of variance model results comparing changes from baseline to 4 weeks or 8 weeks. Groups are as follows: OP = overweight/placebo, OT = overweight/treatment, NP = normal weight/placebo and NT = normal weight/treatment.

**Table 4:** Inter-Group Analysis Comparing the Paired Treatment to PlaceboGroups.

	**Group OP vs. Group 0T**		**Group NP vs. Group NT**	
	**4 weeks**	**8 weeks**	**4 weeks**	**8 weeks**

LAI	<0.0001	<0.0001	<0.0001	<0.0001

CRP	<0.05	<0.001	NS	NS

ESR	NS	NS	<0.05	NS

Weight	<0.01	<0.001	<0.05	<0.01

BMI	NS	NS	NS	NS

Based on analysis of variance F-statistics, P-values are for comparisons between overweight placebo (OP) and overweight treatment (OT) groups and between normal weight placebo (NP) and normal weight treatment (NT) groups at 4 and 8 weeks. NS = not significant.

Comparison of normal weight groups (NT vs. NP) also showed a significant decrease in LAI scores in the treatment compared to the placebo group at 4 weeks (7.6 ± 1.9 vs 10.3 ± 1.5) and 8 weeks (7.7 ± 1.4 vs 9.9 ± 0.9 (both p < 0.0001). For Group NT there were statistically significant decreases in LAI scores compared to baseline at 4 weeks (-3.7 ± 2.1) and 8 weeks (-3.7 ± 1.7) (both p < 0.0001). There was also a difference between the 4 and 8 week time periods (p = 0.012). For Group NP there were significant differences between baseline and 4 weeks (-1.4 ± 2.8; p = 0.019) and between baseline and 8 weeks (-1.9 ± 2.1; p < 0.0001). No significant difference was found between the 4 and 8 week time periods.

Multivariate analysis showed a treatment effect for NP 06-1 in both the overweight and normal weight groups (p < 0.0001 and 0.003, respectively), with no difference in the effect between the overweight and normal weight groups (weight by treatment interaction).

### Biomarkers of Inflammation

The mean total CRP measurements at baseline for the four groups ranged from 0.8 to 1.3 mg/L (Table 5). There were no significant differences between the groups at baseline. Comparison of overweight groups showed a significant difference in CRP between OT and OP groups at 4 and 8 weeks (p < 0.05 and p < 0.001, respectively). For Group OT, there were significant decreases in CRP at 4 weeks (-0.35 ± 0.2; 28%) and 8 weeks (-0.62 ± 0.2; 49%) compared to baseline (both p < 0.001). There were smaller, but significant, changes from baseline for Group OP. Comparison of normal weight groups showed no significant differences in CRP between treatment and placebo. For Group NT there were significant decreases in CRP over time; at 4 weeks (-0.31 ± 0.2; 27%) and 8 weeks (-0.51 ± 0.2; 44%) compared to baseline (both p < 0.001). In contrast there were no significant changes from baseline for Group NP. There were no significant changes in ESR in any of the groups (Table 5).

**Table 5:** Markers of Inflammation: CRP & ESR.

**Group** Variable	Baseline	4 weeks	8 weeks	Δ 0–4 wks	Δ 0–8 wks
**OP**					

CRP (mg/L)	1.19 ± 0.26	1.02 ± 0.27	1.08 ± 0.25	-0.17 ± 0.2*	-0.11 ± 0.07*

ESR (mm/h)	13.6 ± 2.5	13.5 ± 2.0	13.6 ± 1.7	-0.10 ± 2.4	0.0 ± 2.4

**OT**					

CRP (mg/L)	1.33 ± 0.20	0.95 ± 0.11	0.68 ± 0.14	-0.35 ± 0.2*	-0.62 ± 0.2*

ESR (mm/h)	12.7 ± 0.9	13.0 ± 2.1	12.9 ± 1.6	0.31 ± 2.4	0.38 ± 1.8

**NP**					

CRP (mg/L)	0.76 ± 0.19	0.76 ± 0.19	0.68 ± 0.18	0.04 ± 0.1	-0.08 ± 0.1

ESR (mm/h)	12.5 ± 1.4	12.8 ± 1.7	12.8 ± 1.3	0.30 ± 1.0	0.30 ± 1.1

**NT**					

CRP (mg/L)	1.15 ± 0.22	0.84 ± 0.13	0.64 ± 0.50	-0.31 ± 0.2*	-0.51 ± 0.2*

ESR (mm/h)	13.1 ± 1.2	13.5 ± 1.7	13.3 ± 0.9	0.36 ± 2.5	0.18 ± 1.3

Values at baseline, 4 weeks and 8 weeks are listed as means ± standard deviation. Mean changes (Δ) ± standard deviation after 4 weeks and 8 weeks compared to baseline are listed in the last two columns. A negative value indicates a decrease in value and a positive number represents an increase in value. Values* are significant comparisons (<0.05) based on analysis of variance model results comparing changes from baseline to 4 weeks or 8 weeks. Groups are as follows: OP = overweight/placebo, OT = overweight/treatment, NP = normal weight/placebo and NT = normal weight/treatment.

### Weight/BMI

In Group OT, the subjects lost a significant amount of weight and their BMI was reduced compared to the start of the study (p < 0.001 at 4 and 8 weeks) (Table 6). They lost an average of 2.5 kg (5.5 pounds, 3.1% of body weight) after 4 weeks and an average of 4.2 kg (9.2 pounds, 5.1% of body weight) after 8 weeks. There were smaller, but significant, changes in weight and BMI from baseline for Group OP. Comparisons of weight loss between overweight groups revealed that the treatment group lost on average 3.7 times as much weight as the placebo group at 8 weeks (9.2 pounds versus 2.5 pounds) (p < 0.001). However, in comparison, the changes in BMI between the two overweight groups were not significant.

**Table 6:** Body Weight, BMI.

**Group**	Baseline	4 weeks	8 weeks	Δ 0–4 wks	Δ 0–8 wks
**OP**					

Weight (kg)	85.0 ± 12.7	83.9 ± 12.6	83.8 ± 12.2	-1.1 ± 0.96*	-1.2 ± 1.3*

BMI (kg/m^2^)	31.1 ± 4.2	30.7 ± 4.2	30.7 ± 4.1	-0.4 ± 0.59*	-0.4 ± 0.32*

**OT**					

Weight (kg)	81.7 ± 9.3	79.2 ± 9.1	77.5 ± 8.3	-2.5 ± 1.09*	-4.2 ± 1.24*

BMI (kg/m^2^)	31.1 ± 3.7	30.2 ± 3.7	29.5 ± 3.4	-0.9 ± 0.11*	-1.6 ± 0.11*

**NP**					

Weight (kg)	67.1 ± 3.6	66.8 ± 3.8	66.5 ± 3.8	-0.3 ± 0.60	-0.6 ± 0.34*

BMI (kg/m^2^)	24.0 ± 1.6	23.9 ± 1.6	23.8 ± 1.6	-0.1 ± 0.05	-0.2 ± 0.04*

**NT**					

Weight (kg)	67.0 ± 9.7	66.5 ± 8.9	65.8 ± 8.7	-0.5 ± 1.08	-1.2 ± 1.3*

BMI (kg/m^2^)	24.8 ± 2.6	24.7 ± 2.3	24.4 ± 2.3	-0.1 ± 0.05	-0.4 ± 0.08*

Values at baseline, 4 weeks and 8 weeks are listed as means ± standard deviation. Mean changes (Δ) ± standard deviation after 4 weeks and 8 weeks compared to baseline are listed in the last two columns. A negative value indicates a decrease in value and a positive number represents an increase in value. Values* are significant comparisons (<0.05) based on analysis of variance model results comparing changes from baseline to 4 weeks or 8 weeks. Groups are as follows: OP = overweight/placebo, OT = overweight/treatment, NP = normal weight/placebo and NT = normal weight/treatment.

In Group NT there was a significant decrease in weight and BMI at 8 weeks (p < 0.05) compared to baseline. At 8 weeks, this group lost an average of 1.18 kg (2.6 pounds, 1.8% of body weight). Group NP also had a significant loss of weight and BMI after 8 weeks compared to the start of the study (p < 0.01). Comparisons of weight loss between normal weight groups revealed that the treatment group lost on average 1.5 times as much weight as the placebo group at 8 weeks (2.6 pounds versus 1.7 pounds) (p < 0.01). There was no significant difference in change in BMI between the normal weight treatment and placebo groups.

## Discussion

LAI scores (algofunctional indices) have been validated for use in assessing the severity of osteoarthritis of the hip and knee [9]. LAI scores, which incorporate points for pain, maximum distance walked and activities of daily living, typically range from 1 to 14. Scores of 1 to 4 indicate a minor handicap while a score of 14 indicates an extremely severe handicap. Clinical studies used to assess the potential benefits of drugs on osteoarthritis typically start with subjects who have scores of 9 to 11 (SD 2.3 to 3.8). Upon administration of non-steroidal anti-inflammatory drugs (NSAIDS) for one or two weeks, those scores usually decrease by 2.5 to 4 points: decreases of approximately 25 to 40%. In this study, the groups had mean scores within the range of 11 to 13 (SD 1.2 to 2.4) at baseline, which indicates a very severe handicap from osteoarthritis of the knee. After 8 weeks there was a decrease of 3.7 ± 2.1 points in the normal weight treatment group and a decrease of 5.4 ± 2.2 points in the overweight treatment group. The degree of improvement observed with NP 06-1 is comparable to that reported for NSAIDS.

CRP is an acute phase protein that is produced by the liver during an inflammatory reaction. Serum CRP levels are often higher in those with osteoarthritis and are associated with joint pain, as well as the severity and progression of the disease [10,11]. Recent studies have indicated that the correlation between CRP levels and osteoarthritis involves other variables besides the disease state [12]. Body weight has been determined to influence CRP levels due to mechanical effects, as well as the production of inflammatory mediators by adipocytes. A systematic review of the literature covering weight reduction in obese subjects diagnosed with osteoarthritis of the knee concluded that the disability associated with the disease could be significantly improved with a loss of over 5.1% body weight [13]. In contrast with the improvement in disability, loss of body weight did not necessarily predict relief from pain. Another systematic review reported that weight loss in the general adult population is associated with reductions in CRP levels. This analysis concluded that for each 1 kg of weight loss, the mean change in CRP level was decreased by 0.13 mg/L (weighted Pearson correlation, r = 0.85) [14]. A study investigating the correlation between CRP and osteoarthritic disease progression found that subjects with inflammatory infiltrates within the synovial membrane had a mean CRP level of 4.7 ± 5.0 mg/L, while those without infiltrates had a mean CRP level of 1.7 ± 3.6 [11].

In the present study, the mean levels of CRP at baseline for the four groups ranging from 0.76 to 1.3 mg/L indicated a relatively low level of inflammation. In spite of this, there was a reduction in CRP for the overweight participants with treatment compared to placebo. There was no comparative treatment effect for the normal weight participants. However there was an effect of treatment over time for both the overweight and normal weight groups. The overweight treatment group experienced a weight loss of 5.1% after 8 weeks. The normal weight subjects also lost body weight, but to a lesser degree. It appears from current literature, that the decreases in CRP in this study may have been influenced by loss in body weight [13,14]. Also the reduction in weight may have had an influence in the reduction in LAI scores [13].

NP 06-1 contains standardized amounts of berberine, a constituent of the Phellodendron extract, and PMFs from citrus. Both of these components have demonstrated anti-inflammatory activity. Plant extracts containing berberine have traditionally been used in rheumatic and other chronic inflammatory diseases [15]. In support of the traditional use, extracts containing berberine have demonstrated anti-inflammatory activity in animal models [16,17]. In vitro studies indicate that berberine inhibits the production of inflammatory mediators prostaglandin E_2 _and interleukin (IL)-12 [18,19]. Berberine also reduced the levels of expression of mRNA for pro-inflammatory cytokines including tumor necrosis factor (TNF)-alpha, IL-6, CRP and haptoglobin [20].

Berberine has also been shown to reduce body weight in mice and rats [21]. Histological studies indicated that berberine reduced adipocyte size but not cell number. Measurements of metabolic gene expression in adipose and muscle tissue indicated that berberine up regulated genes involved in energy expenditure and down regulated the expression of genes involved in lipogenesis. *In vitro *studies determined that berberine increased AMP-activated protein kinase (AMPK) activity in 3T3-L1 adipocytes and L6 myotubes, as well as reduced lipid accumulation in 3T3-L1 cells. In addition, berberine reduced secretion of leptin, which regulates appetite and energy metabolism, from 3T3-L1 adipocytes.

Citrus PMFs have been reported to exhibit anti-inflammatory activity *in vivo *and *in vitro*. PMFs regulated levels of adipocytokines through suppression of TNF-alpha, interferon-gamma, IL-1 beta and IL-6 expression [22]. PMFs also suppressed TNF-alpha expression by monocytes, perhaps due to inhibition of phosphodiesterase activity [23].

This pilot study has a number of weaknesses. The most obvious weakness is the number of dropouts. There was no clear evidence of difference or bias in the demographics of the dropouts compared to those who stayed in the study; however, there was insufficient information to run an analysis of the two groups. Care must be taken with respect to dropouts when a full blown study is conducted. We also acknowledge that we have no information on patient compliance with treatment protocol.

In summary, it appears from the results of this pilot study that NP 06-1 may benefit subjects with osteoarthritis of the knee through an anti-inflammatory action as well as through a loss of body weight. In addition to increased osteoarthritis-related disability, obesity is correlated with an array of conditions associated with a high risk of diabetes, hypertension and/or cardiovascular disease called metabolic syndrome [24]. Symptoms of metabolic syndrome include abdominal obesity, dyslipidemia and elevated blood pressure and insulin resistance.

We have previously reported that administration of NP 06-1 is associated with a general improvement in lipid levels, with reductions in triglycerides and low-density lipoprotein (LDL)-cholesterol and an increase in high-density lipoprotein (HDL)-cholesterol [7]. In that same report, we also reported treatment-related decreases in blood pressure and a decrease in fasting glucose levels in an overweight population. Thus additional studies are indicated to explore the use of NP 06-1 for osteoarthritis and for metabolic syndrome.

## Conclusion

NP 06-1 has been shown in a placebo-controlled, randomized, double-blind pilot clinical study to offer several potential health benefits in normal and overweight subjects with osteoarthritis of the knee. These potential benefits include improvement of knee joint pain/flexibility as measured by the LAI scores and a reduction in inflammation as measured using CRP levels. Treatment-induced weight loss may have been a contributing factor to these improvements. NP 06-1 was administered to the trial participants in a dose of 4 capsules (1,480 mg) per day for 8 weeks without significant adverse events.

## Competing interests

This study was sponsored by Next Pharmaceuticals. Mr. Garrison was President and Miss Dolnick are employed by Next Pharmaceuticals. Dr. Chambliss and Mr. Kothari were compensated as consultants to Next Pharmaceuticals

## Authors' contributions

All authors were involved in the design of the study, as well as analysis and interpretation of the data. The study was carried out by Dr. Oben, the Principal Investigator and Miss Enonchong, his assistant. All authors read and approved the final manuscript.
